# Acute Myocardial Infarction Followed by Cerebral Hemorrhagic Infarction in Polycythemia Vera: Case Report and Literature Review

**DOI:** 10.3389/fcvm.2021.660999

**Published:** 2021-08-30

**Authors:** XiangSen Shao, ZhuoTing Liu, ChunChang Qin, Fei Xiao

**Affiliations:** ^1^Department of Cardiology, The First Affiliated Hospital of Chongqing Medical University, Chongqing, China; ^2^Department of Neurology, The First Affiliated Hospital of Chongqing Medical University, Chongqing, China

**Keywords:** acute myocardial infarction, polycythemia vera, cerebral infarction, thrombus, diagnosis

## Abstract

A 60-year-old man presented to our emergency room with severe chest pain. Based on the electrocardiogram and elevated serum troponin T levels, acute coronary syndrome was suspected. Coronary angiography revealed total occlusion of the middle of the left anterior descending coronary artery. However, blood cell count abnormalities were not of concern. Twelve days later, the patient developed hemorrhagic infarction in the right parieto-occipital lobe. Acute coronary syndrome and cerebral hemorrhagic infarction were primarily caused by thrombus formation due to polycythemia vera (PV), based on the presence of increased blood consistency on admission. PV was diagnosed after bone marrow biopsy and genetic testing. The patient was treated with descending cell and antiplatelet therapy. Our case highlights the importance of the urgent identification of PV. When acute myocardial infarction occurs in patients with no significant risk factors for cardiovascular disease, blood routine abnormalities should be paid close attention to. If PV was diagnosed as early as possible, thrombotic and hemorrhagic complications could be prevented in the early stages.

## Introduction

A coronary artery plaque is a common cause of acute myocardial infarction (AMI), but some patients may result from other diseases such as polycythemia vera (PV). Therefore, rapid and effective identification of the cause of patients with AMI has important clinical significance. AMI due to PV is often accompanied by blood cell count abnormalities and embolism of other organs, which may be ignored in the early stages. Definitive diagnosis usually requires bone marrow biopsy and genetic examination. Most patients with PV with early intervention have a good prognosis, but the lack of early recognition may cause irreversible accidents.

## Case Report

**Figure 1:**
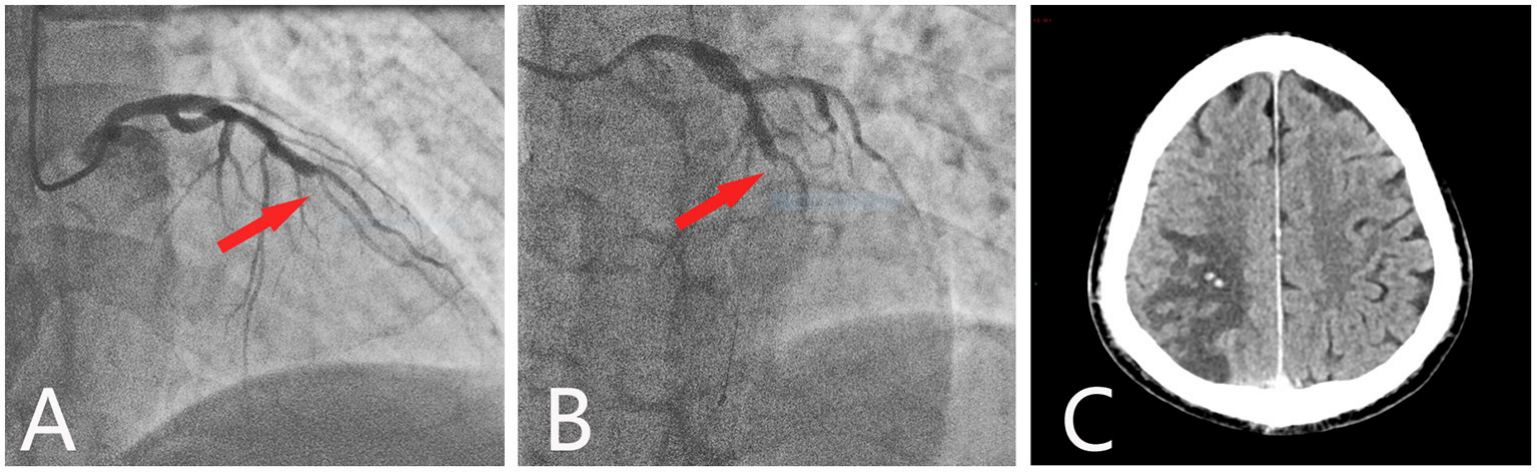
Coronary angiography and cerebral CT. Coronary angiography showed a total occlusion at the mid-left anterior descending branch (red arrow). **(A)** Cranial view. **(B)** Left cranial view. **(C)** Axial cerebral CT showed hypodensity in the right parietooccipital lobe, with two spotted hyperdense areas.

A 60-year-old man presented to the emergency department with chest pain lasting for 2 days. Electrocardiography revealed ST-segment elevation in leads V1–V4 as well as elevated levels of serum troponin T (5.68 ng/mL; normal range, <0.14 ng/mL) and a creatine kinase-myocardial band (33.73 U/L; normal range, <25 U/L), indicating AMI. Routine blood tests showed a mildly increased erythrocyte level (6.05 × 10^9^/L; normal range, 3.5–9.5 × 10^9^/L) and a significantly increased leukocyte level (20.56 × 10^9^/L; normal range, 3.5–9.5 × 10^9^/L) and platelet count (643 × 10^9^/L; normal range, 100–300 × 10^9^/L). However, his hemoglobin level was normal (171 g/L; normal range, 130–175 g/L) (Table 1). Emergency coronary angiography showed total obstruction of the middle of the left anterior descending coronary artery (Figures 1A,B). Therefore, leukocyte elevation was initially considered to be caused by stress after myocardial ischemia. After receiving 300 mg aspirin and 180 mg ticagrelor, a stent was successfully placed, and antiplatelet therapy with oral aspirin (100 mg per day) and ticagrelor (90 mg twice daily) was administered. He had no conventional risk factors for cardiovascular disease. Twelve days later, the patient suddenly developed dizziness, diplopia, and visual distortion. Cerebral computed tomography (CT) revealed a hemorrhagic infarction in the right parieto-occipital lobe (Figure 1C). The craniocervical CT angiogram (CTA) was normal. Blood routine test indicated mildly increased levels of hematocrit (55.4%; normal range, 40–50%), leukocytes (12.12 × 10^9^/L; normal range, 3.5–9.5 × 10^9^/L), and hemoglobin (183 g/L; normal range, 130–175 g/L) and significantly increased platelet count (1,128 × 10^9^/L; normal range, 100–300 × 10^9^/L). A blood electrolyte test showed an elevated potassium concentration (5.9 mmol/L; normal range, 3.5–5.5 mmol/L), and intravenous calcium gluconate was used to reduce blood potassium. However, in the next few days, blood routine examinations showed significantly increased levels of erythrocytes, hemoglobin, hematocrit, and leukocyte and platelet count (Table 1). Thus, bone marrow biopsy was performed, which demonstrated the proliferation of all three cell lineages. Bone marrow aspirate smears revealed active hyperplasia of bone marrow, and the ratio of G/E (granulocytes to erythrocytes) was 1.65. The proportion of granulocyte was increased, accounting for 56%, and granulocytes account for 34%. There were > 200 megakaryocytes. There were no common risk factors of atherosclerotic vascular diseases for the patient, including smoking, hypertension, diabetes, hyperuricemia, hyperhomocysteinemia, hyperlipidemia, and atrial fibrillation, etc.

**Table 1:** Blood routine results of the patient during hospitalization.

**Day of admission**	**Erythrocytes (10^**12**^/L)**	**Hemoglobin (g/L)**	**Hematocrit (%)**	**Platelet (10^**9**^/L)**	**Leucocytes (10^**9**^/L)**
1st	6.05	171	52.6	643	20.56
2nd	5.96	171	52.3	641	17.00
3rd	5.09	150	45.4	691	11.63
10th	5.53	159	48.5	940	11.95
12th	6.24	183	55.4	1,128	12.12
14th	6.44	187	58.2	1,192	15.63
15th	6.80	196	61.0	1,315	15.85
16th	6.54	193	58.2	1,101	14.94
17th	6.47	187	57.6	1,076	11.94
18th	6.33	184	56.9	1,049	10.94

Since hematocrit and hemoglobin levels were significantly increased and bone marrow biopsy showed proliferation of all three lineage cells, PV was strongly suspected. Gene testing confirmed a positive *JAK2*^*V617F*^ mutation. The patient refused venous exsanguination. Patients with thrombosis aged >60 years should be treated with either hydroxyurea or interferon for descending cell therapy. He was then treated with hydroxyurea (500 mg) twice daily and interferon (30 μg) once daily. Since the patient was still required to take antiplatelet drugs to prevent thrombosis and myocardial and cerebral infarctions, the patient was only administered ticagrelor every 12 h. After 3 months of treatment, his blood routine returned to normal.

## Discussion

In this case, the patient had few risk factors for cardiovascular disease, and he was admitted because of acute chest pain. Coronary angiography revealed focal single-vessel disease, and other arteries were patented. During a 12-day hospital stay, the patient's red leucocyte and platelet counts remained high. We initially considered that the elevated leucocyte and platelet counts resulted from a secondary reaction from AMI, but its continued increase was inconsistent with our hypothesis. Subsequently, craniocervical CT was performed because of dizziness, diplopia, and visual distortion and revealed a cerebral hemorrhagic infarction; however, the craniocervical CTA was normal. These findings suggest that thrombus, rather than atherosis, can cause both cerebral and myocardial infarctions. The results of his blood routine, bone marrow biopsy, and genetic analysis met the diagnostic criteria for PV. Therefore, the patient developed a thrombus secondary to PV.

Myocardial infarction and stroke are complications of PV (1). However, myocardial infarction and stroke induced by common risk factors could not be explained for the abnormalities of blood routine test. In this patient, stroke occurred 12 days after myocardial infarction with a larger area identified from imaging examination, which could be attributed to the craniocerebral macrovascular disease. However, CTA of the head and neck did not reveal large blood vessel occlusion, stenosis and atherosclerosis. In addition, stroke occurred after 12-day dual therapy with aspirin and clopidogrel, and as a result, the possibility of stroke caused by cerebrovascular disease risk factors has little correlation. PV may cause sequential thrombosis in multiple organs. A previous study reported the cerebral and coronary vascular occlusive disorders in the patients (2).

Characterized by clonal proliferation of abnormal hematopoietic stem cells, PV is a Philadelphia chromosome-negative chronic myeloproliferative neoplasm. PV is diagnosed when an unexplainable increase in the hemoglobin and hematocrit levels, in addition to a *JAK2* gene mutation, and a decrease in the erythropoietin level are noted (3). *JAK2* mutation results in the substitution of phenylalanine to valine at V617F of the *JAK2* protein, further activating tyrosine kinase expression and promoting the proliferation of hematopoietic precursors (4). Common PV complications include thrombosis and bleeding.

Fagher et al. showed that the platelet count in patients with AMI was significantly lower (5). Due to thrombosis in myocardial infarction and the participation of various inflammatory factors, many platelets are consumed, which results in a decrease in the number of platelets in circulation, which often leads to platelet maturation disorders. This was inconsistent with the laboratory findings of this case.

In a study of 1,213 patients with PV, thrombosis occurred in 19% of patients, of whom 21.7% patients had coronary thrombosis (6). Meanwhile, Hosoya et al. also showed that myocardial infarction is a common complication of PV: the incidences of myocardial infarction and ischemic stroke in patients with PV are 0.32 and 0.53 events/100 persons per year, respectively (1). Due to myelodysplasia, most patients with PV initially show abnormal blood test values, such as thrombocytosis, which may increase the risk of arterial thrombosis (7). Therefore, the platelet count and hematocrit level of myocardial infarction secondary to PV are always higher than those of a simple one, and this cannot be explained by myocardial infarction. In addition to the aforementioned clinical findings, splenomegaly and increases in serum lactate dehydrogenase and neuron-specific enolase levels can be observed in patients with PV (8).

Okabe et al. (9) reported a case of myocardial infarction, even after proper stent placement, and many thromboses reformed on the stent, accompanied by significant elevation of the platelet count and hematocrit level. Bone marrow aspiration revealed bone marrow hyperplasia. This patient was clinically diagnosed with PV. Anticoagulant treatment and phlebotomy procedures were performed during hospitalization, along with the administration of hydroxyurea (1,500 mg/day) for 1 year. Additionally, Cengiz et al. (10) retrospectively collected patients who were diagnosed with PV and presented with acute coronary syndrome. Of these, blood cell count abnormalities such as thrombocytosis were noted, considered as an inflammatory response, while some cases had missed or delayed diagnosis. They also highlighted the importance of blood cell count abnormalities in patients with AMI, especially in the absence of atherosclerotic coronary artery lesions. Although there are several case reports of PV with myocardial infarction, our patient is a rare case of myocardial infarction followed by cerebral stroke.

The exact pathogenesis of thrombosis in PV remains unclear. Some studies attribute it to large numbers of abnormal red blood cells infiltrating the arterial wall, resulting in increased blood viscosity or hyperviscosity syndrome. *JAK2* mutations are present in nearly 95% of patients with PV, and patients with *JAK2* mutations have a high incidence of thrombotic events (11). Excessive activation of the JAK/STAT pathway and platelet turnover are associated with thrombosis (12). Due to accelerated platelet circulation, immature platelets are released into the blood. These compounds have a powerful hemostatic effect (12). Furthermore, enhanced platelet production of 5-hydroxytryptamine, β-thromboglobulin, and platelet factor 4 due to *JAK2 gene* mutation also increases the risk of thrombosis (3). Compared with *JAK2*^*V617F*^ mutation-negative patients, positive patients have higher white blood cell and platelet counts and an increased incidence of thrombosis (13).

Hemorrhagic thrombocythemia is defined as bleeding, with platelets > 1,000 × 10^9^/L, which can explain the bleeding in patients with PV. When platelet counts are between 1,000 and 2,000 × 10^9^/L, bleeding and thrombosis may occur in response. When platelet counts are below 1,000 × 10^9^/L or above 2,000 × 10^9^/L, hemorrhagic thrombocythemia can occur due to acquired von Willebrand syndrome (14). Bleeding after trauma or surgery in patients with PV is probably associated with erythrocyte fallout resulting from a high hematocrit level and impaired platelet-mediated clot retraction (14). Moreover, the use of low-dose aspirin for thrombosis prevention may increase the bleeding tendency of patients with PV. Pseudohyperkalemia is also found in PV, which is associated with blood clotting and the release of potassium from platelets (15).

The treatment of PV depends on whether the patient is symptomatic or asymptomatic. The recommended drugs used are primarily cytoreductive and anticoagulant therapies, including hydroxycarbamide, aspirin, anagrelide, and interferon-alpha (16). With the development of research, *JAK* inhibitors have been developed for the treatment of myeloproliferative neoplasms, including ruxolitinib and fedratinib. Recent studies by Yang et al. have suggested that selective *JAK2* inhibitors could reduce the incidence of complications related to myelodysplasia by inhibiting the cell signal of hematopoietic stem and progenitor cells. Targeted drugs may eventually become the first-line therapy for patients (17). Venous exsanguination and low-dose aspirin are recommended for patients aged <60 years who have no thrombotic history. For patients aged >60 years or those with a history of thrombosis, hydroxyurea should also be administered for descending cell therapy. Interferons may also be selected.

We suggest that patients with myocardial infarction should be paid attention to the abnormal results of blood routine test, in order to avoid missed diagnosis of several rare but important causes, including PV. Blood routine and coagulation tests are two basic laboratory tests which can be carried out even in many developing countries. However, accurate diagnosis of hematological diseases requires specific hematology and molecular testing platform.

## Data Availability Statement

The original contributions presented in the study are included in the article/supplementary material, further inquiries can be directed to the corresponding author/s.

## Ethics Statement

Written informed consent was obtained from the individual for the publication of any potentially identifiable images or data included in this article.

## Author Contributions

XS and ZL: study concept, acquisition of data and figures, and writing of the manuscript. CQ and FX: study concept and critical revision of manuscript for intellectual content. All authors cared for the patient and contributed to writing of the report.

## Conflict of Interest

The authors declare that the research was conducted in the absence of any commercial or financial relationships that could be construed as a potential conflict of interest.

## Publisher's Note

All claims expressed in this article are solely those of the authors and do not necessarily represent those of their affiliated organizations, or those of the publisher, the editors and the reviewers. Any product that may be evaluated in this article, or claim that may be made by its manufacturer, is not guaranteed or endorsed by the publisher.
